# Surgery for carpal tunnel syndrome in patients with and without diabetes–Is there a difference in the frequency of surgical procedures?

**DOI:** 10.1371/journal.pone.0302219

**Published:** 2024-05-08

**Authors:** Anna-Karin Svensson, Lars B. Dahlin, Mattias Rydberg, Raquel Perez, Malin Zimmerman

**Affiliations:** 1 Department of Hand Surgery, Lund University, Skåne University Hospital, Malmö, Sweden; 2 Department of Translational Medicine–Hand Surgery, Lund University, Malmö, Sweden; 3 Department of Orthopedics, Helsingborg Hospital, Helsingborg, Sweden; 4 Department of Biomedical and Clinical Sciences, Linköping University, Linköping, Sweden; 5 Department of Social Epidemiology, Lund University, Malmö, Sweden; Horus University, EGYPT

## Abstract

Carpal tunnel syndrome (CTS) occurs more often among individuals with diabetes. The aim of this retrospective observational registry study was to examine whether individuals with diabetes and CTS are treated surgically to the same extent as individuals with CTS but without diabetes. Data on CTS diagnosis and surgery were collected from the Skåne Healthcare Register (SHR). A total of 35,105 individuals (age ≥ 18 years) diagnosed with CTS from 2004–2019 were included. Data were matched to the Swedish National Diabetes Register (NDR. Cox regression models were used to calculate the risk of the use of surgical treatment. Of the 35,105 included individuals with a CTS diagnosis, 17,662 (50%) were treated surgically, and 4,966 (14%) had diabetes. A higher number of individuals with diabetes were treated surgically (2,935/4,966, 59%) than individuals without diabetes (14,727/30,139, 49%). In the Cox regression model, diabetes remained a significant risk factor for surgical treatment (PR 1.14 (95% CI 1.11–1.17)). Individuals with type 1 diabetes were more frequently treated surgically (490/757, 65%) than individuals with type 2 diabetes (2,445/4,209, 58%). There was no difference between the sexes and their treatment. The duration of diabetes was also a risk factor for surgical treatment in diabetes type 2, but high HbA1c levels were not. Individuals with diabetes are more likely to be treated surgically for CTS than individuals without diabetes. Individuals with type 1 diabetes are more likely to be treated surgically for CTS than individuals with type 2 diabetes.

## Introduction

Nerve entrapment disorders are common, the most frequently encountered being carpal tunnel syndrome (CTS). In the general population, the prevalence is 2.7–3.8% depending on the definition of diagnosis [1]. There are several risk factors for CTS, including hypothyroidism, pregnancy, menopause, obesity, diabetes mellitus, hand-arm vibration syndrome (HAVS), rheumatoid arthritis, distal radius fractures, and occupational risk factors, such as repetitive motions of the hand [2]. Patients with diabetes have a higher prevalence of CTS than the general population [3–5] due to an increased susceptibility to nerve compression. CTS is the most common of the diagnoses included in the “Diabetic Hand” [6].

Diabetes is a growing global health problem, with prevalence increasing in recent decades in countries at all economic levels. Complications of the diabetic hand lead to personal suffering, reduced quality of life, and financial losses due to medical costs and absence from work and to increased pressure on healthcare resources [1].

The treatment options for CTS are either non-operative, including splinting, cortisone injection, and occupational therapy, or surgical release of the carpal ligament, i.e., carpal tunnel release (CTR) [7]. Most patients experience good symptom relief and functional outcomes in the long- and short-term following CTR. The result after CTR in patients with diabetes has not been so extensively investigated [2]. Earlier studies have suggested that patients with diabetes do not benefit from surgery to the same extent as patients without diabetes [8, 9]. There is also some evidence supporting the view that patients with diabetes are at greater risk of developing post-operative infections and secondary surgery after CTR [10]. However, more studies confirm that patients with diabetes experience the same benefit from surgery as the general population [11–13]. One earlier study, with a different design and population, implied that the patients with diabetes had a lower chance of getting surgery [14]. Thus, our aim was to investigate whether individuals with diabetes and CTS are operated on to the same extent as individuals without diabetes and with CTS in a representative (i.e., the general population in the region of Skåne, Sweden) population, adjusting for age, sex and diabetes severity.

## Methods

### Data sources

All caregivers in the Region of Skåne, including primary and secondary care and public and private caregivers [15], are obliged to register patients in the Skåne Health Care Register (SHR). The SHR was started in 1998 and has been improving ever since, with close to 100% coverage of the consultations assigned a diagnosis [15]. The register is an administrative healthcare database with diagnostic codes (ICD.10-SE code G56.0 or ICD-9 354A) and is the basis for economic reimbursement of the healthcare provider [15]. The SHR, which is validated [15], also registers surgical and procedure codes, type of caregiver, and patient age and sex.

In 1996, the National Diabetes Register (NDR) started with the aim of reducing the burden of diabetes in the future. It is used as a quality indicator of the care provider, and the patient can also input their profile online. A substantial number of parameters and risk factors are collected in the register, including diabetes duration, type of diabetes, medications, HbA1c, blood lipid levels, and body mass index (BMI). Each patient provides written informed consent before they are included in the register and may terminate participation at any time. The coverage was around 88% in 2019 [16]. Data compilation was finished, and data was accessed for research purposes on August 16, 2021. The authors had no access to information that could identify individual participants.

### Study population

The study design is a retrospective cohort study, including the 1.1 million adult individuals resident in the Region of Skåne, in the south of Sweden, 2004–2019 [6, 15]. If an individual received a diabetes diagnosis after their CTS diagnosis, they were considered non-diabetic and were included in the group without diabetes. Type 1 diabetes also includes latent autoimmune diabetes in adults (LADA). Diabetic retinopathy was recorded from the first visit registered in the NDR during the study period.

### Statistics

Normally distributed continuous data are presented as mean ± standard deviation (SD). Nominal variables are presented as numbers and percentages, n (%). We compared people with diabetes to people without diabetes as well as people with type 1 diabetes to people with type 2 diabetes. Since the prevalence of the outcomes was relatively high, we measured the relative associations between the explanatory variables and being surgically treated by prevalence ratios (PRs) rather than by odds ratios [17]. For this purpose, we applied Cox proportional hazards regression models with a constant follow-up time equal to 1 using a robust estimator. We developed two regression models. Model 1 included only the diabetes diagnosis, and model 2 added sex and age. We also performed a stratified analysis in diabetic patients to investigate the effect of glycemic control. Glycemic control was classified into 3 groups: optimal control (HbA1c ≤ 48 mmol/mol), acceptable control (HbA1c 48.1–64 mmol/mol), and poor control (HbA1c >64 mmol/mol), which is an adaptation of the American Diabetes Association and National Institute for Health and Care (NICE) guidelines for glycemic control [18, 19]. Confounders were chosen based on previous literature and the authors’ clinical experience. Sex and age might affect the treatment decision. Longer diabetes duration and higher HbA1c levels might cause more severe peripheral neuropathy and are thereby risk factors for developing CTS due to an increased susceptibility to a nerve entrapment disorder like CTS [6, 20]. The other diabetes-related variables were not deemed as possible confounders but are presented in descriptive statistics to present an overview of the disease burden of the included population.

Stata v18.0 (StataCorp, College Station, TX) was used to conduct the analyses.

### Ethics

Ethical permission was granted by the Swedish Ethical Review Authority (https://etikprovningsmyndigheten.se/en/) (Dnr: 2019–02042; 2021–02029). The study was carried out in accordance with the Declaration of Helsinki. Patients provide written informed consent before inclusion in NDR.

## Results

### Study population characteristics

**Fig 1:**
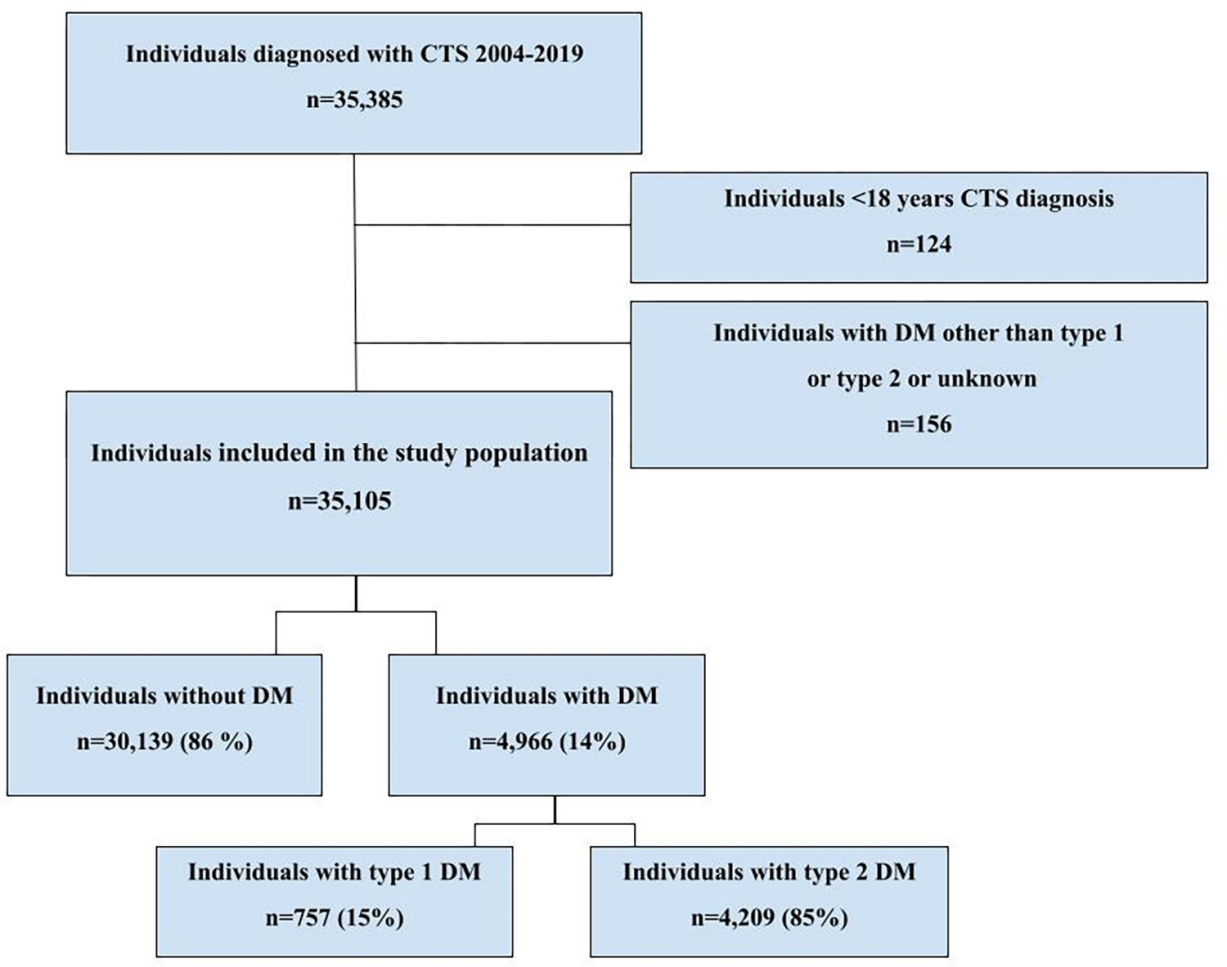
Flowchart illustrating the inclusion process.

From the SHR, we retrieved entries for 35,385 individuals with CTS in primary care or a secondary care facility from 2004–2019. At the time of CTS diagnosis, 35,261 individuals were aged 18 years or older and were included in the study. Using data from the NDR, we crosslinked all data using personal identification numbers and found that 5,122 of the individuals with CTS had diabetes at the time of the CTS diagnosis. Of these, no diabetes type was registered for 80 individuals; 22 individuals had diabetes of a type other than type 1 or type 2, and 58 individuals had an unknown type of diabetes; all of these were excluded from the study population. Ultimately, 35,105 individuals were included in the study: 30,139 with CTS without diabetes and 4,966 with CTS and diabetes. The inclusion process is shown in Fig 1.

### Group characteristics

Individuals with diabetes were older and more often male (Table 1). In the group with diabetes, 757/4,966 (15%) had type 1 diabetes, and 4,209/4,966 (85%) had type 2 diabetes (Fig 1, Table 1). Individuals with type 2 diabetes were older (62±13 years) than individuals with type 1 diabetes (48±14 years). We found no difference regarding sex between the two types of diabetes (Table 1). Retinopathy was more common among individuals with type 1 diabetes than type 2 diabetes (Table 1). Individuals with type 2 diabetes had higher BMI, lower HbA1c, lower creatinine levels, and a shorter diabetes duration than individuals with type 1 diabetes (Table 1).

**Table 1:** Population characteristics, stratified by diabetes.

		No diabetes	Diabetes		
			Type I	Type II	Total
		30,140 (86%)	757 (2%)	4,208 (12%)	4,965 (14%)
Age (years)		52±17	48±14	62±13	60±14
Sex	Men	9,193 (30%)	288 (38%)	1,773 (42%)	2,061 (42%)
	Women	20,947 (70%)	469 (62%)	2,435 (58%)	2,904 (58%)
Surgically treated	No	15,143 (51%)	267 (35%)	1,763 (42%)	2,030 (41%)
	Yes	14,727 (49%)	490 (65%)	2,445 (58%)	2,935 (59%)
Diabetic retinopathy	No		191 (34%)	1,148 (75%)	1,339 (64%)
	Yes		377 (66%)	374 (25%)	751 (36%)
BMI (kg/m^2^)			27.0±4.5	31.5±5.5	30.8±5.6
HbA1c (mmol/mol)			64.5±11.5	53.9±11.6	55.5±12.2
Creatinine (μmol/l)			84.5±62.6	80.1±39.1	80.8±43.5
Diabetes duration (years)			33±15	13±9	16±13

Values presented as n (%) and mean±SD. Data on diabetic retinopathy were missing in 2,876 individuals, BMI in 268 individuals, HbA1c in 9 individuals, creatinine in 75 individuals, and diabetes duration in 489 individuals.

Of all included individuals, 17,662 (50%) had been treated surgically. Among the individuals with diabetes, 2,935/4,966 (59%) were treated surgically compared to 14,727/30,139 (49%) in the group without diabetes (Table 1). More individuals with type 1 diabetes than type 2 diabetes had CTR (65% versus 58%; Table 1). Among those treated surgically, 5,625 (50%) were men and 12,037 (51%) women. There were no differences between the sexes and their treatment, irrespective of whether they had diabetes.

To adjust for age and sex, we performed a Cox regression analysis to assess the prevalence ratio of surgical treatment in the presence of diabetes. Diabetes remained associated with surgical treatment in the adjusted model [PR 1.14 (95% CI 1.11–1.17); Table 2], and diabetes type 1 was even more associated with surgical treatment [PR 1.35 (95% CI 1.28–1.43); Table 2]. HbA1c levels were not significantly associated with CTR, neither in type 1 nor in type 2 diabetes (Table 3). The duration of diabetes was a weak risk factor for surgical treatment in diabetes type 2 (Table 3).

**Table 2:** Model 1. Cox regression measuring associations between diabetes and the risk of being surgically treated for CTS.

	Unadjusted	Adjusted
No diabetes	Reference	Reference
Type I diabetes	1.32 (1.26–1.40)	1.35 (1.28–1.43)
Type II diabetes	1.19 (1.16–1.22)	1.10 (1.07–1.13)

Results are prevalence ratios (PR) with 95% confidence interval (CI). The adjusted model is adjusted for age at diagnosis and sex.

**Table 3:** Model 2. Stratified Cox regression investigating the effect of glycemic control on the risk of being surgically treated for CTS.

Type I diabetes	Optimal control	Reference
	Acceptable control	0.92 (0.73–1.14)
	Poor control	1.00 (1.00–1.00)
	Duration diabetes	1.00 (1.00–1.00)
Type II diabetes	Optimal control	Reference
	Acceptable control	1.00 (0.94–1.06)
	Poor control	1.00 (0.92–1.09)
	Duration diabetes	1.00 (1.00–1.01)

Results are prevalence ratios (PR) with 95% confidence interval (CI). The adjusted model is adjusted for age at diagnosis and sex.

## Discussion

In the present study, which included over 35,000 individuals with CTS, we found a higher rate of surgical treatment for CTS in individuals with diabetes compared to individuals without diabetes. The greatest risk of being treated surgically was found among individuals with type 1 diabetes, independently of sex. Even if the compared groups differed in baseline characteristics, the observed difference remained after adjusting for age and sex. The preconception that surgeons might be less willing to turn to surgical alternatives for their patients with diabetes may, hence, be untrue.

One explanation for the present findings is that individuals with diabetes may present with more severe CTS. Diabetic polyneuropathy (DPN) is a risk factor for developing CTS [21], and it might also, theoretically, be challenging to differentiate between symptoms of CTS and DPN. Furthermore, several studies show that patients with diabetes have worse symptoms before CTR surgery than patients without diabetes [22, 23], which might be one explanation for their being treated surgically to a larger extent. We also found that longer diabetes duration was associated with a higher risk of CTR in type 2 diabetes, probably reflecting the increased prevalence of neuropathy associated with long-standing diabetes [24]. This might also partly explain why individuals with type 1 diabetes are more likely to proceed to surgery since they have had their diabetes longer than their counterparts with type 2 diabetes and are thus at greater risk of developing neuropathy [25]. This is also our clinical experience; patients with long-standing diabetes, as well as patients with type 1 diabetes, present with more severe CTS.

In our study, patients with type 1 diabetes were more likely to be treated surgically than patients with type 2 diabetes. Type 1 diabetes and Type 2 diabetes differ substantially in their pathophysiology regarding the development of neuropathy; hyperglycemia has a greater impact on the development of neuropathy in type 1 diabetes, whereas, in type 2 diabetes, the main driver of neuropathy is deranged blood lipids [26, 27]. However, worse HbA1c control was not associated with a greater risk of CTR in this study, indicating that there may be more mechanisms involved in entrapment neuropathies in people with diabetes.

The prevalence of CTS is higher in individuals with type 1 diabetes [6], and our finding that surgery is more common among individuals with type 1 diabetes might indicate that they suffer from worse neuropathy, which may even be subclinical, than individuals with type 2 diabetes. Another possible explanation is that individuals with diabetes have more frequent healthcare contacts and are more likely to be diagnosed with CTS. This is particularly true for individuals with type 1 diabetes, who are often treated and followed in a specialized care setting, where awareness of complications, such as CTS, is high.

One previous study, using a similar methodology, found that the incidence of CTR was lower in individuals with diabetes compared to those without diabetes [14]. The risk of CTR was also lower in those with diabetes, but the difference disappeared when socioeconomic factors and comorbidities were adjusted for. Even though this study shares some methodological similarities with ours, both being extensive register studies, the populations differ substantially. Reasons for these findings might lie in differences in the healthcare systems, where accessibility to surgery might vary between countries. Misconceptions may exist that individuals with diabetes are less likely to benefit from surgery, with a higher risk of infection [9, 10]. Following CTR, patients with diabetes and neuropathy have more residual symptoms (measured by the QuickDASH), and higher preoperative HbA1c levels are associated with higher (i.e., more symptoms) postoperative QuickDASH scores [22, 28]. Also, electrophysiologic recovery might be less in patients with diabetes [29]. Still, there is now substantial evidence that patients with diabetes have the same outcome after CTR and are content to the same extent as patients without diabetes [2, 11, 12]. In agreement with our results, one Australian observational study found that patients with type 2 diabetes had a higher risk of CTR, but patients with type 1 diabetes were not investigated [30].

When treating patients with CTS, a holistic approach is needed, as prevalence, symptom severity, and recovery are affected by comorbidities, psychological health, and socioeconomic factors–thus, “beyond surgical treatment” [2, 31]. There is also a component of central sensitization in CTS [32], and diabetes itself, especially with an underlying diabetic neuropathy, might cause central sensitization [33]. So, it is possible that even mild CTS may cause more pain symptoms in individuals with diabetes, both due to the increased susceptibility to compression in the peripheral nerve as well as due to increased central sensitization. In analogy, the double crush theory presented by Upton and McComas in 1973 states that the peripheral nerve is more vulnerable to compression if there already is trauma to the nerve somewhere along its course [34]. In CTS and diabetes, diabetes, with its neuropathy, may represent the first crush and CTS the second. This might contribute to an increased need for surgical treatment in the population with diabetes.

In previous studies, the surgery rate for CTS has been reported as 65% for women and 60% for men [35], and 51–81%, depending on sex, age, and geographical region [36]. In two studies [35, 36], CTS diagnoses were obtained only from secondary and tertiary care, i.e., not primary care. Thus, the CTR rate may be higher than in our study, including primary care data, where patients are managed conservatively and may not be referred to secondary care if conservative treatment is successful.

We found no differences in CTR rate between the sexes, either in the whole group or in the group with diabetes. Men might have a greater risk of nerve fiber injury because of the compression, as indicated by worse findings in electrophysiological examinations [37] and a higher risk of diabetic neuropathy [38]. In our study, we do not know in how many patients the CTS diagnosis was investigated using electrophysiological examination. Still, in previous studies carried out by our group, 62% of patients who underwent CTR had had electrophysiology examinations before surgery [28].

## Strengths and limitations

Both the used registers provide reliable data. However, during the first years of operation, the coverage was limited. The NDR was started in 1996 and reached 50% nationwide coverage in 2006, above 86% after 2015 and 88% in 2020, according to the most recent report. As the study is based on registry data, we do not have any data on how the diagnosis of CTS was made, in accordance with other registry studies [39]. We also do not have any clinical data on Body Mass Index (BMI), electrophysiology results, etc.; the latter investigation is not routinely performed in individuals with CTS in Sweden according to guidelines; thus, it is not a gold standard. However, one may anticipate that electrophysiology is more often performed in individuals with diabetes.

The major strengths of this study lie in the large study population, covering all types of care, from primary to secondary, and in the possibility of adequately stratifying for type 1 and type 2 diabetes.

## Conclusions

Individuals with diabetes and CTS are more likely to be treated surgically for CTS than individuals with CTS but without diabetes. Individuals with type 1 diabetes are more likely to be treated surgically for CTS than individuals with type 2 diabetes. Duration of diabetes, but not HbA1c levels, is associated with a greater likelihood of CTR in type 2 diabetes. There was, however, no difference in sex between the surgical and non-surgical groups, irrespective of diabetes diagnosis.
